# Expression Defects of SCN5A Common Polymorphisms S524Y and H558R in the Q1077 Splice Variant Can Be Rescued by Mexiletine

**DOI:** 10.3390/cells15151418

**Published:** 2026-08-05

**Authors:** Rou-Mu Hu, Evelyn J. Song, Carmen R. Valdivia, Isabelle Deschenes, Jonathan C. Makielski, Bi-Hua Tan

**Affiliations:** 1 Heart Center and Beijing Key Laboratory of Hypertension, Beijing Chaoyang Hospital, Capital Medical University, Beijing 100020, China; roumuhu@126.com; 2 Division of Cardiovascular Medicine, Department of Medicine, University of Wisconsin, Madison, WI 53726, USA; crvaldivia@medicine.wisc.edu (C.R.V.); jcmakiel@gmail.com (J.C.M.); 3 Division of Cardiology, Department of Medicine, University of California San Francisco, San Francisco, CA 94403, USA; evelyn.song@ucsf.edu; 4 Department of Physiology and Cell Biology, The Dorothy M. Davis Heart and Lung Research Institute, Frick Center for Heart Failure and Arrhythmia, The Ohio State University, Columbus, OH 43210, USA; isabelle.deschenes@osumc.edu

**Keywords:** SCN5A, splice variant, mexiletine, common polymorphism

## Abstract

**Highlights:**

**What are the main findings?**

- The SCN5A common polymorphisms, S524Y and H558R, exhibit partial expression in the ubiquitous Q1077 splice variant backgrounds.
- The antiarrhythmic drug mexiletine increases the expression of both S524Y and H558R in Q1077 back to wide-type levels.

**What are the implications of the main findings?**

- SCN5A splice-variant context is an important determinant of Na_V_1.5 molecular phenotype.
- The antiarrhythmic drug mexiletine may have a therapeutic modulation of loss-of-function sodium channel defects.

**Abstract:**

The cardiac sodium channel Na_V_1.5, encoded by SCN5A, generates the inward sodium current required for myocardial excitability and impulse conduction. Loss-of-function mutations of Na_V_1.5 have been implicated in inherited arrhythmia syndromes, including Brugada syndrome, progressive cardiac conduction disease, and congenital sick sinus syndrome. The common SCN5A polymorphism H558R has reported minor allele frequencies ranging from 9.2% to 29% across ethnic groups, whereas S524Y has been described in individuals of African ancestry with a minor allele frequency of approximately 3.3%. Two splice variants of human SCN5A, one lacking a glutamine at position 1077 (Q1077del) and one containing Q1077, exist in every human in a 2:1 mRNA transcript ratio. We engineered these two polymorphisms in both backgrounds and reported that when S524Y and H558R were expressed in the Q1077del variant, current densities were normal. In the Q1077 variant, however, the current densities showed a dramatic reduction compared to those in the Q1077del variant or WT-Q1077. We previously reported that incubation with the antiarrhythmic drug mexiletine “rescued” expression deficiencies in the Brugada syndrome. Cells expressing S524Y/Q1077 and H558R/Q1077 were incubated for 48 h with or without mexiletine (500 μM), followed by drug washout before electrophysiological assessment. Mexiletine significantly increased current density for both S524Y/Q1077 and H558R/Q1077 compared with untreated cells, restoring current density to levels comparable to WT-Q1077. Flow cytometry using a FLAG-tagged channel demonstrated that mexiletine-mediated rescue was associated with increased cell-surface expression. The magnitude of the expression defects caused by H558R and S524Y in the Q1077 splice background is similar to that observed with arrhythmia-associated SCN5A mutations, and we show for the first time that the defects for both polymorphisms can be rescued with mexiletine. Although it is unknown whether they result in heightened arrhythmia susceptibility in patients homozygous for the minor allele, our result may have implications for therapy for mutations with loss-of-function phenotypes modified by these common polymorphisms.

## 1. Introduction

The human cardiac sodium channel gene SCN5A encodes the pore-forming α-subunit of the voltage-gated cardiac sodium channel, Na_V_1.5 [1,2]. This channel is responsible for generating a large peak inward Na current (*I*_Na_) which is fundamental to excitability and impulse propagation in working myocardium (atrial and ventricular cells) and special conduction tissue (Purkinje cells and others) [3].

Mutations in SCN5A that cause a spectrum of inherited arrhythmia syndromes, including congenital long QT syndrome subtype 3 (LQT3) [4] and type I Brugada syndrome, are rare [5,6]. Nucleotide variations above a certain threshold allelic frequency, commonly greater than 0.5%, in healthy subjects in a population are called common polymorphisms [7,8]. Among these, H558R is one of the most prevalent SCN5A polymorphisms, appearing with high prevalence (~9–29%) in the general population across all ethnic groups. Clinical studies have further suggested that H558R may be over-represented among patients with Brugada syndrome in the Chinese Han population [9,10,11,12]. Another polymorphism, S524Y, has been described in individuals of African ancestry, with a reported minor allele frequency of 3.3% [9,13]. In addition to genetic variation, SCN5A transcript diversity may influence Na_V_1.5 function. Alternative splicing at the beginning of exon 18 results in inclusion or exclusion of a glutamine residue at position 1077, generating two naturally occurring human SCN5A splice variants: a longer 2016-amino acid isoform containing Q1077 and a shorter 2015-amino acid isoform lacking this residue, designated Q1077del [10,14]. Messenger RNA for both isoforms has been detected in human cardiac tissue, with Q1077del predominating at an approximate 2:1 ratio relative to the Q1077-containing transcript [10,15]. Despite the relative abundance of the Q1077del isoform in human hearts, most prior functional studies of SCN5A variants have been performed in the longer Q1077 background [16]. We previously demonstrated that the functional effects of the common polymorphisms S524Y and H558R are profoundly modified by SCN5A splice-variant context, with S524Y and H558R in the Q1077 background showing a dramatic reduction in current density or “loss of function” [17]. In addition, we have shown that the class Ib antiarrhythmic agent mexiletine can rescue expression-deficient Brugada syndrome-associated SCN5A mutations, including G1743R and R1512W [18,19]. On the basis of these observations, we hypothesized that the expression defect of the common polymorphisms S524Y and H558R in the Q1077 variant can be modulated by mexiletine and tried to explore the mechanisms underlying mexiletine rescue of the expression defective common polymorphisms.

## 2. Methods

### 2.1. Site-Directed Mutagenesis and Heterologous Expression

Mutant constructs [S524Y (c.1571C>A, rs1805126) and H558R (c.1673A>G, rs1805124)] were generated by site-directed mutagenesis in the Q1077 splice variant of the human cardiac voltage-gated sodium channel SCN5A/hNa_V_1.5 cloned into the pcDNA3 vector. Approximately 5 × 10^5^ cells from a transformed human embryo kidney cell line (HEK-293, ATCC, Manassas, VA, USA) were seeded on a 60 mm-diameter plate (Falcon, New York, USA) with 3 mL of culture medium a day before the transfection. Culture medium was MEM complete medium containing Minimum Essential Medium (MEM, Thermo Fisher, Waltham, MA, USA), 10% fetal bovine serum (FBS, HyClone, VWR, Wayne, PA, USA), 2 mM L-glutamine, 0.1 mM MEM nonessential amino acid solution, 1 mM MEM pyruvate solution, 10,000 units penicillin, and 10,000 g streptomycin. The transfections were performed with Superfect (Qiagen, Valencia, CA, USA) according to the manufacturer’s recommended protocol. We added 1.5 μg cDNA per plate. A GFP protein was cotransfected (at 1:5) as a marker to identify the transfected cells.

### 2.2. Standard Electrophysiological Measurements

At 48 h after transient transfection and incubation in the presence or absence of mexiletine, *I*_Na_ was measured using a standard whole-cell patch clamp method at room temperature, 22–24 °C. The extracellular (bath) solution contained 140 mM NaCl, 1.8 mM CaCl_2_, 0.75 mM MgCl_2_, 4 mM KCl and 5 mM HEPES, with pH adjusted to 7.4 using NaOH. The pipette solution contained 120 mM CsF, 20 mM CsCl, 2 mM EGTA, and 5 mM HEPES, with pH adjusted to 7.4 using CsOH. Patch electrodes were fabricated from borosilicate glass using a P-87 puller (Sutter Instrument Co., Novato, CA, USA) and heat-polished with an MF-83 microforge (Narishige, Tokyo, Japan). When filled with recording solution, electrode resistances ranged from 1.0 to 2.0 MΩ. Voltage-clamp recordings were performed using an Axopatch 200B amplifier (Axon Instruments, Foster City, CA, USA) and controlled with pClamp software version 10.2. Series resistance was typically compensated by approximately 80%. The *I*_Na_ was normalized by cell capacitance to obtain current density. After the rupture of the membrane, we performed activation, inactivation, recovery from inactivation, and intermediate inactivation in sequence within 5 min. Standard voltage-clamp protocols for functional characterization were as follows. (1) Activation was measured for steps between −120 mV and +60 mV in 10 mV increments from a holding potential of −140 mV. This protocol was applied to measure peak *I*_Na_ density and activation midpoint. The line represents a fit to the Boltzmann function: Na^+^ conductance (*G*_Na_) = [1 + exp(*V*_1/2_ − *V*)/*k*]^−1^, where *V*_1/2_ and *k* are midpoint and slope factor (as an index of voltage control, all factors were >4), respectively, and *G*_Na_ = *I*_Na(norm)_/(*V* − *V_rev_*), where *V_rev_* is reversal potential and *V* is membrane potential. (2) Steady-state inactivation was measured using a two-step protocol from a holding potential of −140 mV with a 1 s conditioning pulse between −150 mV and 0 mV, followed by a step to 10 mV. The line represents a fit to the Boltzmann function: *I*_Na_ = *I*_Na-max_ [1 + exp(*V*_c_ − *V*_1/2_)/*k*]^−1^, where *V*_1/2_ and *k* stand for midpoint and slope factor, respectively, and *V*_c_ = membrane potential. (3) Recovery from inactivation was measured using a two-pulse protocol in which a conditioning step of 1 s to 0 mV inactivated *I*_Na_, followed by a test pulse to 0 mV after a recovery period of Δt at a recovery potential of −140 mV. The recovery time course was best fit with two exponentials: normalized *I*_Na_ = [A_f_ exp(−t/τ_f_ )]+ [A_s_ exp(−t/τ_s_)], where t = recovery time interval, τ_f_ and τ_s_ = fast and slow time constant, respectively, and A_f_ and A_s_ = fractional amplitude of the fast and slow recovery component, respectively. (4) Development of intermediate inactivation was determined by a two-pulse protocol with a variable duration conditioning step at −60 mV from 1 to 60 s, followed by a 20 ms recovery interval to allow recovery from fast inactivation, and then a test depolarization to −10 mV [17].

### 2.3. Flow Cytometry

For flow-cytometry experiments, a FLAG epitope (DYKDDDDK) was inserted into the Domain I S1-S2 extracellular linker of SCN5A, which places the epitope on the outside of the cells. HEK-293 cells were transiently transfected with FLAG-tagged WT-Q1077, S524Y/Q1077, or H558R/Q1077 constructs. After 48 h of incubation in the presence or absence of 500 μM mexiletine, transfected cells were harvested by incubation with 0.5 mM EDTA-PBS for 10 min at 37 °C. Cells were then washed with staining medium consisting of RPMI 1640 supplemented with 1 mM EDTA (pH 7.4), 3% fetal calf serum, and 0.02% sodium azide. Cells were incubated with FITC-conjugated anti-FLAG M2 antibody (1 μg/mL, Sigma-Aldrich, Saint Louis, MO, USA) in staining medium at 4 °C, followed by washing with PBS containing 1 mM EDTA (pH 7.4) and 1% fetal calf serum. 7AAD was added to exclude the dead cells after the staining. Stained cells were analyzed using a FACSCanto flow cytometer (BD Biosciences, San Jose, CA, USA), as previously described [20,21,22]. FLAG-tagged channel expression was quantified by FlowJo v11 software. The single staining control was used for accurate fluorescence compensation to exclude any spillover between the 7AAD and FITC channels. The debris and doublets were excluded by gating, and 7AAD-negative cells were used for detection of the channel surface expression. The fluorescence value obtained from the control samples, i.e., the cells transfected with the empty vector (vector only), was subtracted as a background from each test sample. The surface expression level of the nucleotide variant and WT channels was measured at the same gate, and their levels were obtained in the three independent experiments and statistically analyzed.

### 2.4. Statistical Analysis

Data are presented as mean ± standard error of the mean (SEM). Statistical comparisons between two groups were performed using Student’s t test, whereas comparisons among multiple groups were analyzed by analysis of variance (ANOVA). *p* < 0.05 after Tukey correction was considered statistically significant.

## 3. Results

### 3.1. Current Expression and Mexiletine Rescue for S524Y and H558R in the Q1077 Background

**Figure 1:**
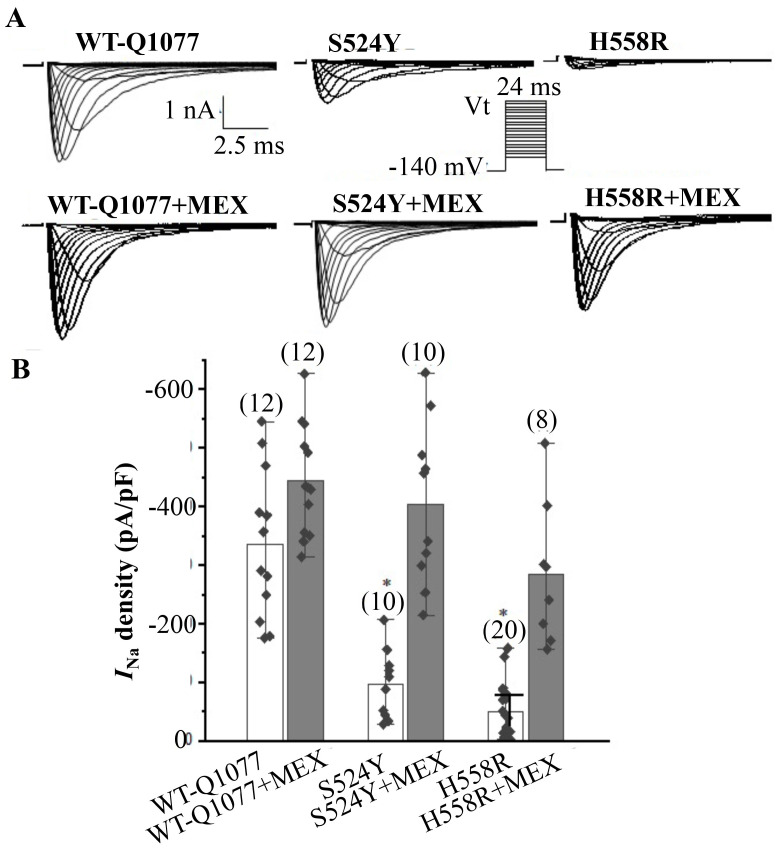
Mexiletine (MEX) rescue of the expression defective polymorphisms, S524Y and H558R in Q1077 background. (**A**) Representative whole-cell current traces from S524Y/Q1077 and H558R/Q1077 channels with or without mexiletine (MEX) treatment, shown in comparison with WT-Q1077 channels. (**B**) Summary of Na+ current (*I*_Na_) density in S524Y, H558R, and WT with and without MEX. The current amplitude was normalized to the membrane capacitance for each cell. * *p* value < 0.005 indicates the *I*_Na_ density was significantly different compared with WT in Q1077 background.

Current densities for the WT and polymorphic channels in the Q1077 background were assessed 48 h after transfection with equal amounts appropriate cDNA and incubation with and without 500 μM mexiletine. *I*_Na_ density was calculated as peak *I*_Na_ normalized to cell capacitance. Mean *I*_Na_ density for the WT, S524Y, and H558R channels were compared for experiments performed on the same day in order to reduce variability. Representative examples of macroscopic *I*_Na_ traces for the WT, S524Y and H558R channels with and without mexiletine are shown in Figure 1A, with summary data given in Figure 1B and Table 1. We found that the current densities without mexiletine incubation were significantly reduced for S524Y and H558R compared to WT-Q1077 (Figure 1B and Table 1). Then we tested the effect on expression of S524Y/Q1077 and H558R/Q1077 after 48 h incubation in mexiletine (500 μM). When studied after the washout of mexiletine, S524Y/Q1077 had dramatically increased *I*_Na_ density of −404 ± 91 pA/pF (*n* = 10) with levels reaching nearly WT levels, compared to no treatment (−97 ± 29 pA/pF, *n* = 10, *p* < 0.005) (Figure 1B and Table 1). H558R/Q1077 also had significantly increased expression levels to −284 ± 87 pA/pF (*n* = 8) compared to no treatment −49 ± 17 pA/pF (*n* = 20, *p* < 0.001) (Figure 1B and Table 1).

### 3.2. Voltage-Dependent Gating Properties of S524Y and H558R in the Q1077 Background

**Figure 2:**
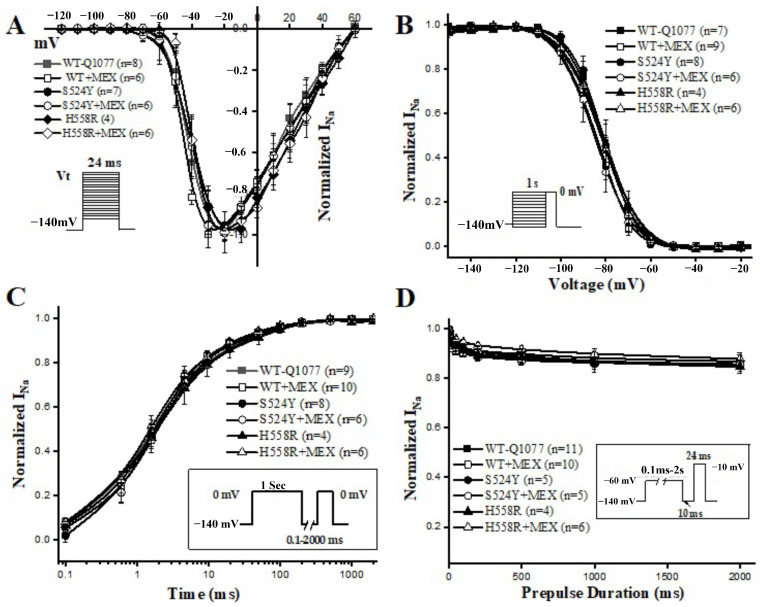
Voltage-dependent gating for S524Y, H558R and WT channels in Q1077 with and without MEX incubation. (**A**) Voltage-dependence of activation for S524Y, H558R and WT channels in Q1077 with and without MEX incubation. The voltage-clamp protocol is shown in the inset. *I*_Na_ was normalized to peak current. (**B**) Steady-state availability from inactivation for S524Y, H558R, and WT-Q1077 with and without MEX incubation. The voltage-clamp protocol is shown in the insert. Normalized peak *I*_Na_ measured in the second step was plotted for the corresponding conditioning potential in the first step. (**C**) Recovery from inactivation for S524Y, H558R, and WT-Q1077 with and without MEX incubation, and with time displayed on a logarithmic scale to highlight the early phase of recovery. Voltage protocol is shown in the insert with normalized *I*_Na_ plotted against the recovery time between the two pulses. (**D**) Intermediate inactivation for S524Y, H558R and WT-Q1077 with and without MEX incubation.

The voltage-dependent gating properties of S524Y and H558R channels with and without mexiletine pre-incubation are compared with those of WT channels in Figure 2; summary data for the fitted kinetic parameters are shown in Table 1. The activation midpoints were not different for S524Y/Q1077 with and without mexiletine and H558R/Q1077 with mexiletine compared to WT-Q1077 (*p* > 0.05) (Figure 2A and Table 1). For steady-state inactivation, the midpoints were not significantly different for S524Y/Q1077 with and without mexiletine and H558R/Q1077 with mexiletine compared with WT-Q1077 (Figure 2B and Table 1). For the recovery from inactivation, no significant differences were observed for S524Y/Q1077 and H558R/Q1077 with and without mexiletine compared to WT-Q1077 (Figure 2C and Table 1). For intermediate inactivation, the S524Y/Q1077 and H558R/Q1077 showed no difference with and without mexiletine treatment compared with WT-Q1077 (Figure 2D).

### 3.3. Cell Surface Expression of S524Y and H558R Channel Protein in Q1077 Background with and Without Drug Treatment

**Figure 3:**
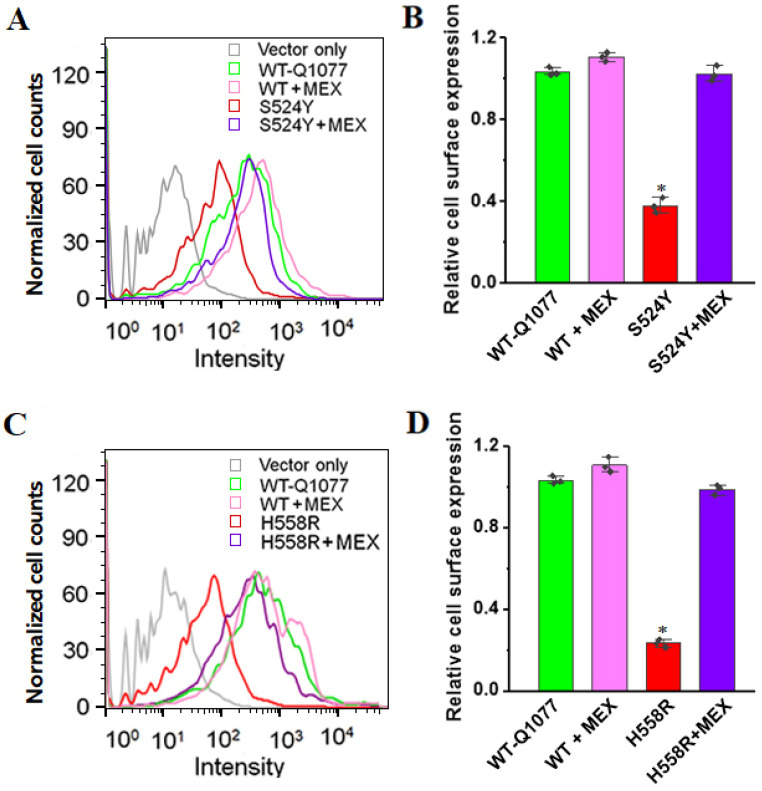
Flow cytometric analysis of cell surface expression of the FLAG-tagged S524Y, H558R and WT channels in Q1077 background. (**A**) The FLAG-tagged channel expression of FLAG-tagged WT-Q1077, FLAG-tagged WT + MEX, FLAG-tagged S524Y, FLAG-tagged S524Y + MEX and Vector only. (**B**) Quantification of the percentage of cells with detectable plasma membrane expression of FLAG-tagged WT-Q1077, FLAG-tagged WT + MEX, FLAG-tagged S524Y and FLAG-tagged S524Y + MEX. * *p* < 0.005, compared with WT-Q1077. (**C**) The FLAG-tagged channel expression of FLAG-tagged WT-Q1077, FLAG-tagged WT + MEX, FLAG-tagged H558R, FLAG-tagged H558R + MEX and Vector only. (**D**) Quantitation data to demonstrate the percentage of counted cells which the plasma membrane expression of FLAG-tagged WT-Q1077, FLAG-tagged WT + MEX, FLAG-tagged H558R and FLAG-tagged H558R + MEX. * *p* < 0.001, in comparison with WT-Q1077.

We used flow cytometry to detect the cell surface expression of FLAG-tagged WT-Q1077, S524Y/Q1077, and H558R/Q1077. Little expression of Na channels at the cell surface were noted for both S524Y/Q1077 and H558R/Q1077 compared with WT-Q1077 (Figure 3A,C). These results suggest that the current expression defect is caused by a reduction in channel expression at the cell surface. We further explored whether drug treatment could affect the cell surface expression. Consistent with the electrophysiological data shown in Figure 1, prior incubation with mexiletine markedly increased surface expression of both S524Y/Q1077 and H558R/Q1077 channels (Figure 3B,D).

## 4. Discussion

In the present study, we confirmed [11] that the common polymorphisms S524Y and H558R exhibited significantly decreased peak *I*_Na_ in the Q1077 splice background. The new findings are that the antiarrhythmic drug mexiletine improved the expression of both S524Y and H558R channels and restored the *I*_Na_ density after incubation to nearly WT levels. These data suggest that the loss-of-function with these common polymorphisms is likely caused by an expression defect that can be rescued by mexiletine. Furthermore, flow cytometry data suggest that the mechanism for this restoration was to increase expression of the channels at the cell surface.

Historically, functional studies of loss-of-function SCN5A mutations associated with arrhythmia syndromes have often been performed in a single splice-variant background, named Q1077del. However, in common polymorphisms and mutations introduced into both splice variants there were significant differences in expression level, channel gating, and even the success of therapeutic rescue for a given variant [9,11,13,16,17]. Several prior observations illustrate the importance of this interaction between primary sequence variation and splice background. Wang et al. reported that an in-frame deletion, delAL586-587, and two missense variants, R680H and V1951L, produced increased persistent *I*_Na_ only when expressed in the Q1077del splice variant [23]. Similarly, we previously showed that the SCN5A variant S1787N significantly augmented late *I*_Na_ only in the Q1077del background [24]. In contrast, other disease-associated variants appear to manifest expression defects preferentially in the Q1077-containing isoform. Specifically, we identified splice-dependent reductions in channel expression for the SCN5A mutations G1406R [15] and R1512W when expressed in the Q1077 background [19]. In prior work, we examined six polymorphisms (R481W, S524Y, H558R, P1090L, S1103Y, and R1193Q) and demonstrated that each exhibited splice background-dependent functional phenotypes [17]. In particular, S524Y and H558R produced marked reductions in sodium current density when expressed in the Q1077 background. The remaining polymorphisms, R481W, P1090L, S1103Y, and R1193Q, showed splice-dependent alterations in activation, inactivation, or recovery from inactivation. Together, these observations underscore the importance of evaluating both common polymorphisms and putative arrhythmia-associated SCN5A variants in both splice variant backgrounds.

The antiarrhythmic drug mexiletine has been reported to restore the loss of SCN5A function caused by arrhythmia mutations first reported by Valdivia et al. for M1766L [25,26]. Mexiletine also rescued a mixed biophysical phenotype of the cardiac sodium channel arising from the SCN5A mutation N406K [20]. In another study, the expression defect of the rare variant/Brugada mutation R1512W was rescued by mexiletine in both the Q1077del and the Q1077 background [19]. In contrast, mexiletine only partially restored the current density of V1378M but had no effect on the current density of A124D [19]. Nonetheless we hypothesized that mexiletine might also be able to rescue the expression defect of the SCN5A common polymorphisms S524Y and H558R in the Q1077 background and our results demonstrate that mexiletine does restore current levels.

Mexiletine is a class Ib antiarrhythmic agent that interacts with Na_V_1.5 through the local anesthetic binding site located within the sixth transmembrane segment of domain IV [27]. Beyond its acute use-dependent sodium channel blocking effects, mexiletine has been proposed to facilitate membrane expression of select trafficking-deficient SCN5A variants [28,29]. Moreau et al. suggested that the binding of mexiletine to V1378M channels within the endoplasmic reticulum may promote proper folding or conformational stabilization, thereby enabling export of channels that would otherwise be retained intracellularly [30]. In contrast, variants were located too distantly from the local anesthetic binding site for mexiletine to be effective, such as the A124D mutation in the N-terminus. This suggests that the effectiveness of mexiletine to rescue trafficking defects may depend on the location of the mutation. Both S524Y and H558R reside within the cytoplasmic linker between domains I and II. This region remains incompletely resolved in available structural models of SCN5A/Na_V_1.5, indicating that the functional significance of variants in this region remain unknown [31].

What is the mechanism for the loss of function for S524Y and H558R in the Q1077 background, and the rescue by mexiletine? Mutations in SCN5A may cause “loss-of-function” by several general mechanisms [32,33]. First, variants that introduce premature termination codons may result in truncated transcripts or nonsense-mediated decay, thereby preventing the production of functional channel protein. Second, full-length channel protein may be synthesized but fail to traffic efficiently to the plasma membrane, resulting in reduced surface channel expression. Third, channels may reach the cell surface but exhibit impaired function because of altered gating kinetics, abnormal voltage dependence, defective pore conductance, or other biophysical abnormalities [15]. Previously we demonstrated by flow cytometry that the rescues of mutations R1512W [13] and N406K [15] were accompanied by the reversal of defective channel expression at the cell surface. In this study we show that similarly, this may be the mechanism of rescue for the expression defect of common polymorphisms H558R and S524Y (Figure 3). These data favor the idea that the loss of function for H558R and S524Y in Q1077del is a trafficking defect, and that mexiletine corrects the defect. We speculate that the trafficking defect is caused by a misfolded protein, and that mexiletine binding somehow corrects this. However, the detailed structural mechanisms for how a single amino acid addition at position 1077 interacts with other structures to cause this effect, and how mexiletine causes a rescue, remain unknown.

Several limitations should be acknowledged. First, this study used a “minimalist” heterologous expression system, which may not fully reflect Na_V_1.5 behavior in native cardiomyocytes. In cardiac tissue, SCN5A functions within a larger macromolecular complex that includes β subunits and other interacting proteins that may modulate channel trafficking, localization, and gating; these components were not co-expressed in our model. In addition, the constructs used here lack endogenous intronic, promoter, and regulatory elements that may influence SCN5A expression in cardiomyocytes. Finally, the mexiletine concentration used in these experiments exceeds clinically achievable levels. Thus, these findings identify a potential biophysical in a heterologous system, but further studies in cardiomyocyte-based models will be necessary to define the full pathogenic mechanism and assess the therapeutic relevance of pharmacologic rescue.

## 5. Conclusions

In conclusion, this study characterized the electrophysiological function and cell surface expression of the SCN5A common polymorphisms, S524Y and H558R, in the Q1077 splice variant backgrounds and confirmed a partial expression defect with both polymorphisms in the Q1077 background. The antiarrhythmic drug mexiletine increased the expression level of both S524Y and H558R in Q1077 back to WT levels as demonstrated by current measurements and channel cell surface expression. These findings provide further evidence that SCN5A splice-variant context is an important determinant of Na_V_1.5 molecular phenotype, with potential implications for genotype–phenotype interpretation and therapeutic modulation of loss-of-function channel defects.

## Figures and Tables

**Table 1:** Voltage-dependent gating properties of each group in heterologous expression system.

	Density	Activation	Inactivation	Recovery		
Samples	pA/pF	V_1/2_ (mV)	V_1/2_ (mV)	τ_f_ (ms)	τ_s_ (ms)	A_s_ (%)
WT-Q1077	−336 ± 64 (12)	−43.0 ± 1.8 (8)	−81 ± 2.9 (7)	1.8 ± 0.3	35 ± 5.3	24 ± 1.4 (9)
WT + MEX	−445 ± 52 (12)	−44.6 ± 0.9 (6)	−83 ± 2.7 (9)	2.0 ± 0.2	36 ± 5.0	20 ± 1.5 (10)
S524Y/Q1077	−97 ± 29 * (10)	−42.8 ± 2.4 (7)	−81 ± 2.3 (8)	1.6 ± 0.2	32 ± 4.5	26 ± 1.7 (8)
S524Y + MEX	−404 ± 91 (10)	−43.0 ± 4.4 (6)	−84 ± 3.2 (6)	2.1 ± 0.3	39 ± 8.4	21 ± 1.6 (6)
H558R/Q1077	−49 ± 17 * (20)	−40.7 ± 4.0 (4)	−81 ± 2.2 (4)	1.6 ± 0.3	31 ± 5.2	25 ± 1.8 (4)
H558R + MEX	−284 ± 87 (8)	−41.0 ± 3.0 (6)	−82 ± 1.3 (6)	1.7 ± 0.2	31 ± 4.5	23 ± 1.3 (6)

Values are presented as mean ± SEM, with the number of experiments shown in parentheses. Parameters were derived by fitting data from individual experiments to the appropriate model equations. For Boltzmann fits, V_1/2_ represents the midpoint voltage of activation or inactivation. * *p* value < 0.005 indicates the *I*_Na_ density was significantly different compared with WT in Q1077 background.

## Data Availability

The original contributions presented in this study are included in this article. The data used and/or analyzed during the current study are available from the corresponding author upon reasonable request.

## References

[1] Bennett P.B., Yazawa K., Makita N., George A.L. (1995). Molecular mechanism for an inherited cardiac arrhythmia. Nature.

[2] Amin A.S., Asghari-Roodsari A., Tan H.L. (2010). Cardiac sodium channelopathies. Pflug. Arch..

[3] Zaytseva A.K., Kulichik O.E., Kostareva A.A., Zhorov B.S. (2024). Biophysical mechanisms of myocardium sodium channelopathies. Pflug. Arch..

[4] Wilde A.A.M., Amin A.S. (2018). Clinical Spectrum of SCN5A Mutations: Long QT Syndrome, Brugada Syndrome, and Cardiomyopathy. JACC Clin. Electrophysiol..

[5] Ackerman M.J., Clapham D.E. (1997). Ion channels—Basic science and clinical disease. N. Engl. J. Med..

[6] Denham N.C., Pearman C.M., Ding W.Y., Waktare J., Gupta D., Snowdon R., Hall M., Cooper R., Modi S., Todd D. (2019). Systematic re-evaluation of SCN5A variants associated with Brugada syndrome. J. Cardiovasc. Electrophysiol..

[7] Hull J., Thomson A.H. (1998). Contribution of genetic factors other than CFTR to disease severity in cystic fibrosis. Thorax.

[8] Wilkins M.R., Roses A.D., Clifford C.P. (2000). Pharmacogenetics and the treatment of cardiovascular disease. Heart.

[9] Ackerman M.J., Splawski I., Makielski J.C., Tester D.J., Will M.L., Timothy K.W., Keating M.T., Jones G., Chadha M., Burrow C.R. (2004). Spectrum and prevalence of cardiac sodium channel variants among black, white, Asian, and Hispanic individuals: Implications for arrhythmogenic susceptibility and Brugada/long QT syndrome genetic testing. Heart Rhythm..

[10] Makielski J.C., Ye B., Valdivia C.R., Pagel M.D., Pu J., Tester D.J., Ackerman M.J. (2003). A ubiquitous splice variant and a common polymorphism affect heterologous expression of recombinant human SCN5A heart sodium channels. Circ. Res..

[11] Viswanathan P.C., Benson D.W., Balser J.R. (2003). A common SCN5A polymorphism modulates the biophysical effects of an SCN5A mutation. J. Clin. Investig..

[12] Lizotte E., Junttila M.J., Dube M.P., Hong K., Benito B., De Zutter M., Henkens S., Sarkozy A., Huikuri H.V., Towbin J. (2009). Genetic modulation of brugada syndrome by a common polymorphism. J. Cardiovasc. Electrophysiol..

[13] Shuraih M., Ai T., Vatta M., Sohma Y., Merkle E.M., Taylor E., Li Z., Xi Y., Razavi M., Towbin J.A. (2007). A common SCN5A variant alters the responsiveness of human sodium channels to class I antiarrhythmic agents. J. Cardiovasc. Electrophysiol..

[14] Modell S.M., Lehmann M.H. (2006). The long QT syndrome family of cardiac ion channelopathies: A HuGE review. Genet. Med..

[15] Tan B.H., Valdivia C.R., Song C., Makielski J.C. (2006). Partial expression defect for the SCN5A missense mutation G1406R depends on splice variant background Q1077 and rescue by mexiletine. Am. J. Physiol. Heart Circ. Physiol..

[16] Gellens M.E., George A.L., Chen L.Q., Chahine M., Horn R., Barchi R.L., Kallen R.G. (1992). Primary structure and functional expression of the human cardiac tetrodotoxin-insensitive voltage-dependent sodium channel. Proc. Natl. Acad. Sci. USA.

[17] Tan B.H., Valdivia C.R., Rok B.A., Ye B., Ruwaldt K.M., Tester D.J., Ackerman M.J., Makielski J.C. (2005). Common human SCN5A polymorphisms have altered electrophysiology when expressed in Q1077 splice variants. Heart Rhythm..

[18] Valdivia C.R., Tester D.J., Rok B.A., Porter C.B., Munger T.M., Jahangir A., Makielski J.C., Ackerman M.J. (2004). A trafficking defective, Brugada syndrome-causing SCN5A mutation rescued by drugs. Cardiovasc. Res..

[19] Hu R.M., Song E.J., Tester D.J., Deschenes I., Ackerman M.J., Makielski J.C., Tan B.H. (2021). Expression defect of the rare variant/Brugada mutation R1512W depends upon the SCN5A splice variant background and can be rescued by mexiletine and the common polymorphism H558R. Channels.

[20] Hu R.M., Tester D.J., Li R., Sun T., Peterson B.Z., Ackerman M.J., Makielski J.C., Tan B.H. (2018). Mexiletine rescues a mixed biophysical phenotype of the cardiac sodium channel arising from the SCN5A mutation, N406K, found in LQT3 patients. Channels.

[21] Shikano S., Li M. (2003). Membrane receptor trafficking: Evidence of proximal and distal zones conferred by two independent endoplasmic reticulum localization signals. Proc. Natl. Acad. Sci. USA.

[22] Liu K., Yang T., Viswanathan P.C., Roden D.M. (2005). New mechanism contributing to drug-induced arrhythmia: Rescue of a misprocessed LQT3 mutant. Circulation.

[23] Wang D.W., Desai R.R., Crotti L., Arnestad M., Insolia R., Pedrazzini M., Ferrandi C., Vege A., Rognum T., Schwartz P.J. (2007). Cardiac sodium channel dysfunction in sudden infant death syndrome. Circulation.

[24] Hu R.M., Tan B.H., Tester D.J., Song C., He Y., Dovat S., Peterson B.Z., Ackerman M.J., Makielski J.C. (2015). Arrhythmogenic Biophysical Phenotype for SCN5A Mutation S1787N Depends upon Splice Variant Background and Intracellular Acidosis. PLoS ONE.

[25] Valdivia C.R., Ackerman M.J., Tester D.J., Wada T., McCormack J., Ye B., Makielski J.C. (2002). A novel SCN5A arrhythmia mutation, M1766L, with expression defect rescued by mexiletine. Cardiovasc. Res..

[26] Varian K., Tang W.H.W. (2017). Therapeutic Strategies Targeting Inherited Cardiomyopathies. Curr. Heart Fail. Rep..

[27] Weiser T., Qu Y., Catterall W.A., Scheuer T. (1999). Differential interaction of R-mexiletine with the local anesthetic receptor site on brain and heart sodium channel alpha-subunits. Mol. Pharmacol..

[28] Nakajima T., Dharmawan T., Kawabata-Iwakawa R., Tamura S., Hasegawa H., Kobari T., Ota M., Tange S., Nishiyama M., Kaneko Y. (2021). Reduced current density, partially rescued by mexiletine, and depolarizing shift in activation of SCN5A W374G channels as a cause of severe form of Brugada syndrome. Ann. Noninvasive Electrocardiol..

[29] Nasilli G., Yiangou L., Palandri C., Cerbai E., Davis R.P., Verkerk A.O., Casini S., Remme C.A. (2023). Beneficial effects of chronic mexiletine treatment in a human model of SCN5A overlap syndrome. Europace.

[30] Moreau A., Keller D.I., Huang H., Fressart V., Schmied C., Timour Q., Chahine M. (2012). Mexiletine differentially restores the trafficking defects caused by two brugada syndrome mutations. Front. Pharmacol..

[31] Wagner E., Marras M., Kumar S., Kelley J., Ruff K., Silva J. (2025). Investigating the role of the I-II linker in Nav1.5 channel function. J. Gen. Physiol..

[32] Kataoka N., Imamura T. (2023). Brugada Syndrome: A Comprehensive Review of Fundamental and Electrophysiological New Findings. J. Clin. Med..

[33] Heymans A.B.M., Bianchi L., Volders P.G.A., van der Crabben S.N., Verdonschot J.A.J. (2025). SCN5A Cardiomyopathy: From Ion Channel Dysfunction To Clinical Disease. Curr. Cardiol. Rep..

